# Indications for Tube Feeding in Adults with Muscular Disorders: A Scoping Review

**DOI:** 10.3233/JND-230014

**Published:** 2023-09-08

**Authors:** Marloes Middelink, Nicol C. Voermans, Baziel G.M. van Engelen, Mirian C.H. Janssen, Jan T. Groothuis, Simone Knuijt, Heidi Zweers-van Essen

**Affiliations:** a Department of Gastroenterology and Hepatology – Dietetics, Radboud University Medical Center, Nijmegen, The Netherlands; b Department of Neurology, Donders Institute for Brain, Cognition and Behaviour, Radboud University Medical Center, Nijmegen, The Netherlands; c Department of Internal Medicine, Radboud University Medical Center, Nijmegen, The Netherlands; d Department of Rehabilitation, Donders Centre for Brain Cognition and behaviour, Radboud University Medical Center, Nijmegen, The Netherlands

**Keywords:** Enteral nutrition, dysphagia, pneumonia, aspiration, malnutrition, weight loss, muscular dystrophies, myositis, inclusion body, myopathies, structural, congenital

## Abstract

**Background:**

Eating an adequate diet and maintaining a healthy body weight can be challenging for patients with muscular disorders (MD). Starting tube feeding can have a positive impact on nutritional status, functioning and quality of life. Guidelines on when to start tube feeding in adults with MD are lacking.

**Objective:**

We aim to review the scientific literature on indications to start tube feeding in adults with facioscapulohumeral dystrophy (FSHD), inclusion body myositis (IBM), muscular dystrophy type 1 (DM1), oculopharyngeal muscular dystrophy (OPMD) and congenital myopathies.

**Methods:**

This scoping review was conducted according to the Preferred Reporting Items for Systematic Reviews and Meta-Analyses extension for scoping reviews (PRISMA-ScR) guidelines. Relevant studies were identified in Pubmed, Embase and Cinahl (April 2022). The medical subject headings (MeSH) and text words used were related to FSHD, IBM, DM1, OPMD or congenital myopathies and dysphagia, enteral nutrition or malnutrition.

**Results:**

Of 1046 unique articles, 9 case reports and 2 retrospective case series were included. Indications to start tube feeding were dysphagia, malnutrition/weight loss and respiratory infections (due to aspiration). Percutaneous endoscopic gastrostomy (PEG) tubes were used most often and complications were respiratory failure, problems with the tube itself, accidental tube removal, cutaneous symptoms, digestive symptoms, and peritonitis.

**Conclusion:**

Data on tube feeding in MD is scarce. Indications to start tube feeding were similar across the various MD. We call for more research in this field and suggest to include screening for dysphagia, aspiration and malnutrition in for the treatment of various MD.

## INTRODUCTION

Eating an adequate diet and maintaining a healthy body weight is important for patients with neuromuscular disorders to maintain quality of life However, this challenging due to dysphagia, chewing difficulties, gastro-intestinal symptoms, apathy and fatigue. Malnutrition and weight loss can accelerate disease progression and lead to increased morbidity. Additionally, complications of severe dysphagia, such as pneumonia or obstruction, is a leading cause of death in MD [1]. Starting with (additional) tube feeding may have a positive impact on nutritional status, functioning, prognosis and health related quality of life of MD patients [2]. Our clinical experience is that MD patients often start tube feeding too late.

Surprisingly, for the most common inherited myopathies there are no guidelines on indications for starting tube feeding. The timing to start tube feeding in facioscapulohumeral dystrophy (FSHD), inclusion body myositis (IBM), muscular dystrophy type 1 (DM1), oculopharyngeal muscular dystrophy (OPMD) and congenital myopathies is unknown. Common features in these disorders are progressive muscular weakness, dysphagia and experienced fatigue (Table 1) [3–12]. Ultimately these symptoms can lead to problems with preparing and consuming a healthy diet, resulting in malnutrition [13]. In turn, malnutrition leads to increased muscular weakness and fatigue, in other words: a vicious cycle.

**Table 1:** Characteristics of the MD

MD	Age of onset	Pathogenesis	Symptoms
FSHD [4, 5, 8]	10 – 20	Shortening (type 1) or random mutation (type 2) of the fourth chromosome	Progressive muscle weakness, dysphagia, speech problems
IBM [6]	<50	Immune attack of T-cells to muscle cells	Progressive muscle weakness, dysphagia
DM1 [3, 10, 11]	Mild: <50Adult onset: 12–50Child onset: 1–12Congenital: at birth	Expansion of a CTG repeat	Dysphagia, myotonia, progressive muscle weakness, tongue weakness, obesity, affected cognition, diarhoea
OPMD [7, 9, 12]	40–60	Expansion of GCN repeat in 14th chromosome leading to aggregation of protein	Progressive muscle weakness, ptosis, dysphagia, fatigue
Congenital myopathies	At birth	Differs per myopathy	Hypotonia, muscle weakness, delayed motor development

In the early stages of these MD when symptoms are mild, practical advices on how to adjust eating habits to a healthy diet can improve nutritional status. This advice can comprise for example drinking sips of water during the meal, change the consistency of the foods to a more liquid form or to adjust postural position to optimise the swallowing process. Non-compensatory interventions can exist of oral motor exercises to improve swallowing function and mastication, for example by using chewing gum [14]. However, these are only temporary solutions as the dysphagia progresses. Permanent interventional solutions are a percutaneous endoscopic gastrostomy (PEG) or percutaneous radiologic gastrostomy (PRG) [15]. Because difficulties in eating and keeping an adequate diet develop insidiously and there is no consensus on when to start tube feeding, patients might already be too weak to start tube feeding once initiated. As such, a clear answer regarding the optimal implementation of tube feeding in MD through means of the available literature is warranted.

In contrast to the MD mentioned above, guidelines on nutritional management are available in amyotrophic lateral sclerosis (ALS) and Duchenne muscular dystrophy (DMD). Indications to start tube feeding in ALS and DMD are: dysphagia, weight loss (>10% in six months or >5% in one month), inadequate fluid intake, aspiration (pneumonia), relative decrease in ventilatory capacity, taxing meal duration and fatigue due to eating [16, 17]. Almost all patients with ALS and DMD will need tube feeding in the course of their disease. This creates alertness for nutritional status among clinicians. Since malnutrition is less common in FSHD, IBM, DM1, OPMD and congenital myopathies (although in IBM, DM1 and OPMD, dysphagia is very common), we cannot simply adopt the ALS and DMD guidelines to FSHD, IBM, DM1, OPDM and congenital myopathies.

To gain more insight in the indications to start tube feeding in these MD, we performed a scoping review. We aim to summarize the indications for tube feeding reported in literature for, as well as the type of tubes used, whether the patient lost weight and what complications were observed. We expect that this review will contribute to increased awareness and ultimately better management for patients with FSHD, IBM, DM1, OPMD and congenital myopathy.

## MATERIALS AND METHODS

### Search strategy

This scoping review was conducted according to the Preferred Reporting Items for Systematic Reviews and Meta-Analyses extension for scoping reviews (PRISMA-ScR) guidelines. Relevant studies using medical subject headings (MeSH) and text words related to all types of FSHD, IBM DM1, OPMD, or congenital myopathies and dysphagia, enteral nutrition or malnutrition were identified (Appendix 1). Pubmed, Embase and Cinahl were searched in April 2022 without any search limits. There were no restrictions on publication year, as cases from long ago can still be relevant for this review. The search strategy for Pubmed was created together with a data specialist of our medical library and changed accordingly for the other databases.

### Study selection

First, all duplicates were deleted using Endnote. Next, the remaining papers were screened on title and abstract and selected using Rayyan, a web-tool for systematic reviews. Eligibility criteria were: studies or cases with adult (>18 years) patients with one of the following MD: FSHD, IBM, DM1, OPMD or congenital myopathy, in combination with malnutrition, tube feeding or dysphagia, and the English language. Exclusion criteria were: studies on children, animal studies, overview articles, other (MD) disorders, and an alternative focus of the article. Lastly, full-texts of the selected papers were reviewed for suitability. Reference lists were reviewed for additional publications.

### Outcome measures

The primary outcome measure was the indication to start tube feeding. The secondary outcome measures were type of tube, weight loss before tube placement, and complications after placement of the tube.

## RESULTS

**Fig. 1:**
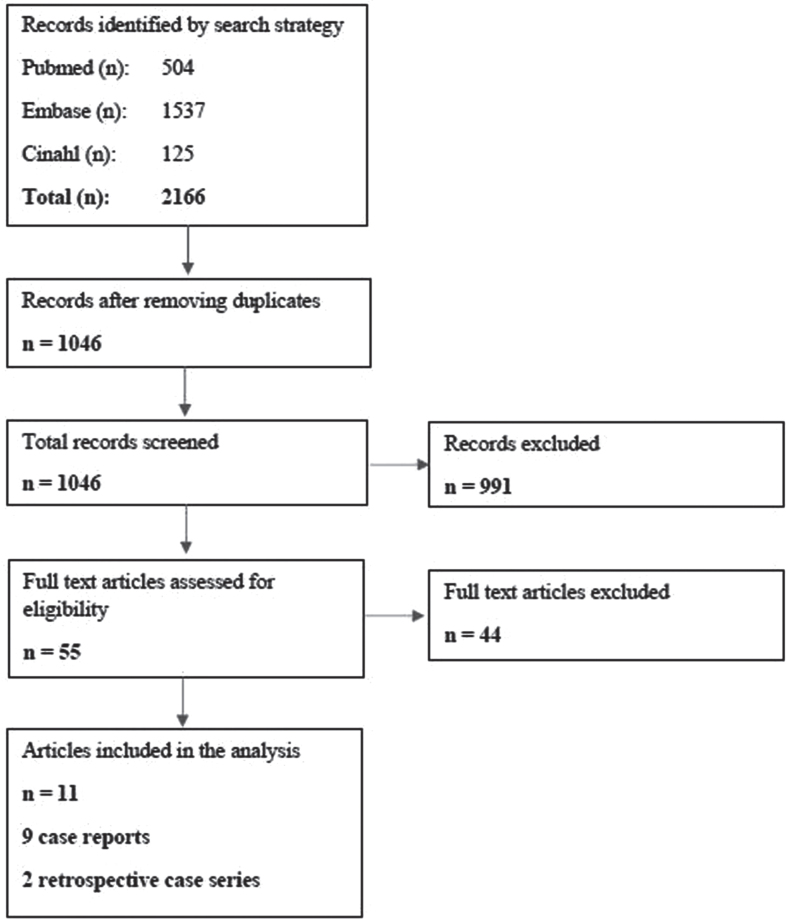
Flow chart of the search strategy.

The search strategy yielded 2166 results (Fig. 1). After deleting duplicates, 1046 articles were screened for title and abstract. After reading 55 full texts, 9 case reports and 2 retrospective case series were included in the analysis. During the assessment for eligibility, articles were excluded because the participants described did not receive enteral feeding. The 9 case studies were only cases of DM1 and OPMD, and the retrospective case series contained cases of FSHD, IBM, and congenital myopathies.

### Case characteristics

Of the nine case reports (two females), four patients had a DM1 diagnosis and five patients had an OPMD diagnosis (Table 2). The DM1 patients had a mean age of 37, while the OPMD patients had a mean age of 73. The two retrospective case series consisted of 26 and 144 patients (Table 2). The 26 patients (20 females) of the first retrospective case series all had an IBM diagnosis and a mean age of 72 at the time of the study. Six of these 26 patients started tube feeding. The second retrospective case series was a mixed population with DMD (*n* = 77), FSHD (*n* = 5), DM1 (*n* = 40), and congenital myopathy (*n* = 24) diagnoses and all started tube feeding. However, this retrospective case series only compared DMD and DM1, because these were the largest groups, revealing no information about the FSHD and congenital myopathy patients.

**Table 2:** Details of tube feeding in the nine case reports

Author, year [reference]	Study design	Number of patients that started tube feeding	Diagnosis	Age (mean)	Sex (M/F)	Indications tube feeding (n)	Disease duration (years)	Type of tube (n)	Weight loss	Complications
Allen &O’Leary, 2018 [28]	Case report	1	CongenitalDM1	21	M	Dysphagia, (Risk for) aspiration	21	NR	NR	Respiratory failure
Bumm, Zenker &Bozzate, 2009 [29]	Case report	1	OPMD	57	M	Acute pneumonia, Progressive dysphagia Malnutrition	0*	PEG	NR	NR
Christopher et al., 2001 [30]	Case report	1	OPMD	83	M	OPMD diagnosis	0*	PEG	No	Respiratory failure, therefore intubated
Fuchs, Hoedemaekers &der Hoeven, 2009 [31]	Case report	1	Adult onset DM1	33	M	Poor gastric retention, Weight loss	0	Gastric-duodenal tube	Yes	Respiratory failure
Hebbar, Webberly, Lunt &Robinson, 2007 [32]	Case report	1	OPMD	76	M	Progressive dysphagia, impossibility to perform a myotomy	0	PEG	Yes	NR
Ishizawa, Okano, Sasaki, Tomioka &Araki, 2014 [33]	Case report	1	Adult-onset DM1	61	M	Dysphagia, Recurrent coughs	4– 8	Abdominal Gastrostomy	NR	NR
Kiel, 1986 [34]	Case report	1	OPMD	80	F	Severe dysphagia	NR	NR	Yes	NR
Malik, Sharma, Moreno &Parcha, 2022 [35]	Case report	1	Adult-onset DM1	34	F	Progressive dysphagia	0	PEG	Yes	Respiratory failure
Perie et al., 1997 [36]	Case series	1 (4.5%)	OPMD	(68)	M	Dysphagai, weight loss, Aspiration pneumonia	11.8	Naso-esophageal tube	Yes	Aspiration pneumonia (died)
Oh, Brumfield, Hoskin, Kasperbauer &Basford, 2008 [37]	Retrospective case series	6 (23%)	IBM	(72)	6/20	Aspiration pneumonia (5)Dysphagia (1)	67 mos after diagnosis	PEG (6)	NR	Aspiration pneumonia, Respiratory failure
Mizuno et al., 2012 [38]^**^	Retrospective case series	144 (100%)	DMD (55%)DM1 (28%)Congenital myopathies (15%)FSHD (3%)	26 (DMD) 55 (DM1) 22/62 (Congenital myopathies) 52 (FSHD) (at time of placement)	NR	Dysphagia (105)Malnutrition (29)Recurrent respiratory infections (21)Trouble tube feeding (11)Digestive symptoms (10)	NR	PEG (118)Laparostomy (17)Unknown (12)	NR	Cutaneous symptoms, Respiratory failure, Problems with tube, Accidental tube removal, Digestive symptoms, Peritonitis

DM1 = Myotonic Dystrophy type 1; DMD = Duchenne Muscular Dystrophy; FSHD = Facioscapulohumeral Dystrophy; IBM = Inclusion Body Myositis; OPMD = Oculopharyngeal Dystrophy; NR = Not Reported; PEG = Percutaneous Endoscopic Gastrostomy; M = Male; F = Female. ^*^Diagnosed when visiting the hospital for dysphagia symptoms. ^**^DMD and DM1 were compared, therefore the information on the tube indications and type, weight loss and complications are only described for these diagnoses.

### Primary outcomes

The case reports showed that the most frequently reported reasons to start tube feeding were: dysphagia (4/9), (risk for) aspiration pneumonia (2/9) and weight loss (2/9) (Table 1). In the first retrospective case series, the indications to start tube feeding were aspiration pneumonia (5/6) and dysphagia (1/6). Most frequently mentioned indications for tube feeding in the second retrospective case series were dysphagia (105/144), malnutrition(29/144) and respiratory infections (21/144) (Table 2). When (risk for) aspiration pneumonia was an indication to start tube feeding, the patient always experienced recurrent aspirations. Dysphagia was severe or progressive, or it was not clear what the severity of the dysphagia was. Severity of malnutrition or amount of weight loss was not mentioned.

### Secondary outcomes

Type of tube Four out of nine patients from the cases received a PEG tube, one an abdominal gastrostomy, one a naso-esophageal tube and one a gastric-duodenal tube. In two cases the type of tube was unspecified (Table 2). In the first retrospective case series all six patients received a PEG tube. In the second retrospective case series PEG tubes were received by 118/144 patients, and laparostomy by 18/144 patients. Of 12 patients it was unknown what type of tube they received (Table 2).

Weight loss In four out of nine cases the patients experienced weight loss before the tube placement, in one case there was no weight loss and in the other four cases this was not reported (Table 2). In both retrospective case series it was not reported whether the patients experienced weight loss before tube placement (Table 2).

Complications After tube placement, patients experienced respiratory failure, problems with the tube itself, accidental tube removal, cutaneous symptoms, digestive symptoms and peritonitis (Table 2). The patients that died during follow up, died of aspiration pneumonia despite the tube feeding (*n* = 9) or respiratory failure (*n* = 1) or an unknown cause (*n* = 5).

## DISCUSSION

Data on tube feeding in FSHD, DM1, IBM, OPMD and congenital myopathies is scarce. The results of this scoping review show that the main reasons to start tube feeding in adults with these MD were and respiratory infections (due to aspiration). Most patients received a PEG tube, but other tubes were also reported. Some patients experienced weight loss before tube placement, but this was not always reported. Complications after tube placement were respiratory failure, problems with the tube itself, accidental tube removal, cutaneous symptoms, digestive symptoms, and peritonitis. Based on the very little findings of this scoping review, we call for more research on tube feeding in adults with MD. We found only 9 cases and 2 retrospective case series, making it impossible to generalize our results to the whole population. We call for more systematically reported research on when patients with MD started tube feeding and also on patients that did not start tube feeding. Dieticians and speech therapist should be involved in this research as they are experts in this field. With such research, recommendations regarding tube feeding in adults with MD can be made and guidelines can be changed accordingly. Until then, clinicians should comply to the general guidelines for MD, but can take dysphagia, aspiration and malnutrition into account as warning signs that a patient could benefit from tube feeding.

The currently available guidelines for the MD of interest contain only limited information on dysphagia, aspiration, and malnutrition. The 2015 consensus guideline on FSHD does not even mention dysphagia, aspiration, or/nor malnutrition [18], although the consequences of orofacial weakness have been reported [19]. The consensus guideline for congenital myopathies mentions the importance of screening for these risk factors in children only. Although it emphasizes the importance of a good transition into adult care, there are no recommendations for the adult care of patients with congenital myopathies [20]. The current guidelines for DM1 only recommend dysphagia screening [21]. Based on the observations in this review, we recommend including the screening of dysphagia, aspiration and malnutrition in revisions of these guidelines.

Because the indications to start tube feeding in the above-mentioned MD are very similar to the indications to start tube feeding in ALS and DMD patients [16, 17], the revision of the guidelines for DM1, OPMD and IBM patients could be informed by the knowledge and experience of the ALS and DMD guidelines. An important difference is that not all patients with FSHD, DM1, OPMD and IBM congenital myopathies will need tube feeding. This underscores the heterogeneity of the diseases and the importance of screening and personalized management in MD. This article calls for more attention on tube feeding in adults with MD. Regular screening of dysphagia, aspiration and malnutrition could be included in the clinical guidelines for patients with all MD and in daily clinical practice to find the patients that could benefit from tube feeding. The articles included in this review did not report which screening tools are most appropriate. A multidisciplinary team including at least a speech-language therapist and dietician is necessary to monitor the patient on all these screening aspects. To enable weight monitoring of the patients, hospitals should have adequate weighing equipment that is suitable for patients that are wheelchair-dependent. It is not feasible to perform difficult or time-consuming tests on each patient at every hospital visit. Therefore, short, standardised questionnaires assessing dysphagia, aspiration and malnutrition should be used as screening tools. The Eating Assessment Tool (EAT-10) is validated and reliable questionnaire for dysphagia. With a score of three or higher, the patient should be referred to a speech-language therapist [22]. The speech therapist can decide whether a video fluoroscopy, fibreoptic endoscopic evaluation of swallowing or manometry is necessary. To screen for malnutrition, Patient Generated Subjective Global Assessment (PG-SGA) could be used. This questionnaire contains questions about weight, nutrition and daily activities and can pick up malnutrition in patients without weight loss as well and is available in multiple languages [23]. Both tools are short questionnaires that can easily be completed before or during hospital visits. It is important to not solely use (loss of) body weight as an indication of malnutrition, as MD patients can have a high nutritional risk without weight loss due to high caloric food choices with low nutritional value and decrease in physical activity [24]. Additionally, sarcopenic obesity is found in multiple MD and related to a greater chance of morbidity and mortality [25, 26]. For diagnosing sarcopenic obesity body composition measurements like Bioelectrical Impedance Analysis (BIA) or Dual-energy X-ray absorptiometry (DXA) are necessary. Dieticians can give guidance to patients with personalised food intake advice based on the nutritional assessment outcomes [27]. After tube placement, a dietician determines the amount of tube feeding and should be consulted with problems as a consequence of the change in diet, such as obstipation and nausea [16].

This review has shed light on the scarcity on research on the indications to start tube feeding in patients with FSHD, IBM, DM1, OPMD and congenital myopathies. A limitation is that cases with poor outcomes that did not start tube feeding were not included. It is not unlikely that these cases would have benefited from an earlier start of tube feeding. Furthermore, some indications might have been missed because they were not included in the search strategy. Also, index of indications was not always mentioned, making it hard to compare severity of dysphagia, malnutrition or pneumonia. In our experience, there are numerous barriers to start tube feeding. However, to date, there is little research on how these barriers evolved and how they can be overcome. This should be investigated in more detail in future research.

## CONCLUSION

Data on indications of tube feeding in patients with MD are generally scarce, apart from a few diseases such as ALS and DMD. The few cases and retrospective case series available show that dysphagia, aspiration and malnutrition are the most important indications to start tube feeding in patients with IBM, DM1, and OPMD. No information was available for FSHD and congenital myopathies. Based on the scarce literature and the experience in our neuromuscular clinic we conclude that more research is necessary to make solid recommendations. Until then, neuromuscular clinics could include screening for dysphagia, aspiration and malnutrition to their regular examination during an outpatient consultation, by means of questionnaires and monitoring weight. Systematic documentation of these data will provide more information on the patients that benefit from tube feeding. By monitoring weight, clinicians can pay attention to (sarcopenic) obesity as well as malnourishment. Screening for dysphagia, aspiration and malnutrition can ensure a timely referral to a speech-language therapist and/ or dietician, and a multidisciplinary approach to the dietary care for this patient. If indicated, tube feeding can be started to prevent malnutrition and aspiration and through this improve the patient’s quality of life and limit disease progression.

## Supplementary Material

Supplementary Material
