# Supplementary material for: Indications for Tube Feeding in Adults with Muscular Disorders: A Scoping Review
Source: J Neuromuscul Dis. 2023 Sep 8;10(5):777–85. doi: 10.3233/JND-230014 (PMC10578233; doi:10.3233/JND-230014)
Supplement: Supplementary Material [file jnd-10-jnd230014-s001.docx]

**Appendix 1:** Search strategy

*Pubmed*

("Muscular Dystrophy, Facioscapulohumeral"[Mesh] OR "Muscular Dystrophy, Oculopharyngeal"[Mesh] OR "Myotonic Dystrophy"[Mesh] OR "Myositis, Inclusion Body"[Mesh]

OR "Myopathies, Structural, Congenital"[Mesh]

*OR*

FSHD[tiab] OR IBM[tiab] OR OPMD[tiab] OR DM1[tiab] OR congenital myopath*[tiab] OR Facioscapulohumeral Muscular Dystroph*[tiab] OR FSH Muscular Dystroph*[tiab] OR Facio-Scapulo-Humeral Dystrophy[tiab] OR Landouzy Dejerine Dystroph*[tiab] OR Inclusion Body Myositis[tiab] OR Inclusion Body Myopath*[tiab] OR Oculopharyngeal Muscular Dystroph*[tiab]

OR Oculopharyngeal Dystroph*[tiab] OR Myotonic Dystrophy Type 1[tiab] OR Myotonic Dystrophies Type 1[tiab] OR Myotonic Dystrophy 1[tiab] OR Myotonic dystrophy type I[tiab] OR Myotonic dystrophy I[tiab] OR Steinert*[tiab] OR Congenital Myotonic Dystroph*[tiab] OR Congenital Structural Myopath*[tiab] OR Congenital myopath*[tiab] OR myotubular myopath*[tiab] OR Rod Myopath* [tiab] OR Nemaline Myopath*[tiab] OR Central Core Myopath*[tiab] OR Central Core Disease[tiab] OR X-Linked Myotubular Myopath*[tiab] OR XLMTM[tiab] OR X-Linked Centronuclear Myopath*[tiab] OR Congenital Fiber Type Disproportion*[tiab] OR Congenital Myopathy with Fiber Type Disproportion*[tiab] OR CFTDM[tiab] OR Centronuclear Myopath*[tiab])

*AND*

(“Enteral Nutrition”[Mesh] OR “Pneumonia, Aspiration”[Mesh] OR “Malnutrition”[Mesh] OR "Deglutition Disorders"[Mesh]

*OR*

Enteral Nutrition[tiab] Enteral Feeding*[tiab] OR Force Feeding*[tiab] OR Tube Feeding[tiab] OR Gastric Feeding Tube*[tiab] OR PRG[tiab] OR PEG[tiab] OR Percutaneous radiologic gastrostomy[tiab] OR Percutaneous endoscopic gastrostomy[tiab] OR Aspiration Pneumonia*[tiab] OR Acid Aspiration Syndrome*[tiab] OR Nutritional Deficienc*[tiab] OR Undernutrition[tiab] OR Undernourish*[tiab] OR Malnutrition[tiab] OR Malnouris*[tiab] OR Deglutition Disorder*[tiab] OR Swallowing Disorder*[tiab] OR Dysphagia[tiab] OR Esophageal Achalasias[tiab] OR Cardiospasm*[tiab] OR Achalasia*[tiab])

*Embase*

(facioscapulohumeral muscular dystrophy/ or myotonic dystrophy/ or oculopharyngeal muscular dystrophy/ or inclusion body myositis/ or central core disease/ or nemaline myopathy/ or centronuclear myopathy/

*OR*

(FSHD or IBM or OPMD or DM1 or congenital myopath* or Facioscapulohumeral Muscular Dystroph* or FSH Muscular Dystroph* or Facio-Scapulo-Humeral Dystrophy or Landouzy Dejerine Dystroph* or Inclusion Body Myositis or Inclusion Body Myopath* or Oculopharyngeal Muscular Dystroph* or Oculopharyngeal Dystroph* or Myotonic Dystrophy Type 1 or Myotonic Dystrophies Type 1 or Myotonic Dystrophy 1 or Myotonic dystrophy type I or Myotonic dystrophy I or Steinert*

or Congenital Myotonic Dystroph* or Congenital Structural Myopath* or Congenital myopath* or myotubular myopath* or Rod Myopath* or Nemaline Myopath* or Central Core Myopath* or Central Core Disease or X-Linked Myotubular Myopath* or XLMTM or X-Linked Centronuclear Myopath* or Congenital Fiber Type Disproportion* or Congenital Myopathy with Fiber Type Disproportion*

or CFTDM or Centronuclear Myopath*).ti,ab,kf.)

*AND*

(enteric feeding/ or percutaneous endoscopic gastrostomy tube/ or exp feeding tube/ or exp aspiration pneumonia/ or percutaneous endoscopic gastrostomy/ or malnutrition/ or dysphagia/ or esophagus achalasia/

*OR*

(Enteral Nutrition or Enteral Feeding* or Enteric Feeding* or Enteric Nutrition or Force Feeding* or Tube Feeding or Gastric Feeding Tube* or PRG or PEG or Percutaneous radiologic gastrostomy or Percutaneous endoscopic gastrostomy or Aspiration Pneumonia* or Acid Aspiration Syndrome* or Nutritional Deficienc* or Undernutrition or Undernourish* or Malnutrition or Malnourish* or Deglutition Disorder* or Swallowing Disorder* or Dysphagia or Esophageal Achalasias or Cardiospasm* or Achalasia*).ti,ab,kf.)

*Cinahl*

((MH "Muscular Dystrophy, Facioscapulohumeral") OR (MH "Muscular Dystrophy, Oculopharyngeal") OR (MH "Myotonic Dystrophy") OR (MH "Myositis, Inclusion Body")

*OR*

TI (FSHD or IBM or OPMD or DM1 or congenital myopath* or Facioscapulohumeral Muscular Dystroph* or FSH Muscular Dystroph* or Facio-Scapulo-Humeral Dystrophy or Landouzy Dejerine Dystroph* or Inclusion Body Myositis or Inclusion Body Myopath* or Oculopharyngeal Muscular Dystroph* or Oculopharyngeal Dystroph* or Myotonic Dystrophy Type 1 or Myotonic Dystrophies Type 1 or Myotonic Dystrophy 1 or Myotonic dystrophy type I or Myotonic dystrophy I or Steinert*

or Congenital Myotonic Dystroph* or Congenital Structural Myopath* or Congenital myopath* or myotubular myopath* or Rod Myopath* or Nemaline Myopath* or Central Core Myopath* or Central Core Disease or X-Linked Myotubular Myopath* or XLMTM or X-Linked Centronuclear Myopath*

or Congenital Fiber Type Disproportion* or Congenital Myopathy with Fiber Type Disproportion*

or CFTDM or Centronuclear Myopath*)

*OR*

AB (FSHD or IBM or OPMD or DM1 or congenital myopath* or Facioscapulohumeral Muscular Dystroph* or FSH Muscular Dystroph* or Facio-Scapulo-Humeral Dystrophy or Landouzy Dejerine Dystroph* or Inclusion Body Myositis or Inclusion Body Myopath* or Oculopharyngeal Muscular Dystroph* or Oculopharyngeal Dystroph* or Myotonic Dystrophy Type 1 or Myotonic Dystrophies Type 1 or Myotonic Dystrophy 1 or Myotonic dystrophy type I or Myotonic dystrophy I or Steinert* or Congenital Myotonic Dystroph* or Congenital Structural Myopath* or Congenital myopath* or myotubular myopath* or Rod Myopath* or Nemaline Myopath* or Central Core Myopath* or Central Core Disease or X-Linked Myotubular Myopath* or XLMTM or X-Linked Centronuclear Myopath*

or Congenital Fiber Type Disproportion* or Congenital Myopathy with Fiber Type Disproportion*

or CFTDM or Centronuclear Myopath*))

*AND*

((MH “Enteral Nutrition”) OR (MH“Pneumonia, Aspiration”) OR (MH “Malnutrition”) OR (MH “Esophageal Achalasia”)

*OR*

TI (Enteral Nutrition or Enteral Feeding* or Enteric Feeding* or Enteric Nutrition or Force Feeding* or Tube Feeding or Gastric Feeding Tube* or PRG or PEG or Percutaneous radiologic gastrostomy or Percutaneous endoscopic gastrostomy or Aspiration Pneumonia* or Acid Aspiration Syndrome* or Nutritional Deficienc* or Undernutrition or Undernourish* or Malnutrition or Malnourish* or Deglutition Disorder* or Swallowing Disorder* or Dysphagia or Esophageal Achalasias or Cardiospasm* or Achalasia*)

*OR*

AB (Enteral Nutrition or Enteral Feeding* or Enteric Feeding* or Enteric Nutrition or Force Feeding* or Tube Feeding or Gastric Feeding Tube* or PRG or PEG or Percutaneous radiologic gastrostomy or Percutaneous endoscopic gastrostomy or Aspiration Pneumonia* or Acid Aspiration Syndrome* or Nutritional Deficienc* or Undernutrition or Undernourish* or Malnutrition or Malnourish* or Deglutition Disorder* or Swallowing Disorder* or Dysphagia or Esophageal Achalasias or Cardiospasm* or Achalasia*))
